# Using the CollaboraKTion framework to report on primary care practice recruitment and data collection: costs and successes in a cross-sectional practice-based survey in British Columbia, Ontario, and Nova Scotia, Canada

**DOI:** 10.1186/s12875-018-0782-x

**Published:** 2018-06-13

**Authors:** Sabrina T. Wong, William Hogg, Fred Burge, Sharon Johnston, Ilisha French, Stephanie Blackman

**Affiliations:** 1 School of Nursing, University of British Columbia, T201 2211 Westbrook Mall, Vancouver, BC V6T 2B5 Canada; 2 Centre for Health Services and Policy Research, University of British Columbia, 201-2206 East Mall, Vancouver, BC V6T 1Z3 Canada; 3 Department of Family Medicine, University of Ottawa, 201-600 Peter Morand Cresc, Ottawa, ON K1G 5Z3 Canada; 4 Montfort Hospital Research Institute, 713 Montreal Rd, Ottawa, ON K1K 0T2 Canada; 5 Department of Family Medicine, Dalhousie University, 5909 Veterans’ Memorial Lane, Abbie J. Lane Building, Halifax, NS B3H 2E2 Canada

**Keywords:** Waiting room, Patient experience, Provider, Engagement, Integrated knowledge translation

## Abstract

**Background:**

Across Canada and internationally we have poor infrastructure to regularly collect survey data from primary care practices to supplement data from chart audits and physician billings. The purpose of this work is to: 1) examine the variable costs for carrying out primary care practice-based surveys and 2) share lessons learned about the level of engagement required for recruitment of practices in primary care.

**Methods:**

This work was part of a larger study, TRANSFORMATION that collected data from three provincial study sites in Canada. We report here on practice-based engagement. Surveys were administered to providers, organizational practice leads, and up to 20 patients from each participating provider. We used the CollaboraKTion framework to report on our recruitment and engagement strategies for the survey work. Data were derived from qualitative sources, including study team meeting minutes, memos/notes from survey administrators regarding their interactions with practice staff, and patients and stakeholder meeting minutes. Quantitative data were derived from spreadsheets tracking numbers for participant eligibility, responses, and completions and from time and cost tracking for patient survey administration.

**Results:**

A total of 87 practices participated in the study (*n* = 22 in BC; *n* = 26 in ON; *n* = 39 in NS). The first three of five CollaboraKTion activities, Contacting and Connecting, Deepening Understandings, and Adapting and Applying the Knowledge Base, and their associated processes were most pertinent to our recruitment and data collection. Practice participation rates were low but similar, averaging 36% across study sites, and completion rates were high (99%). Patient completion rates were similarly high (99%), though participation rates in BC were substantially lower than the other sites. Recruitment and data collection costs varied with the cost per practice ranging from $1503 to $1792.

**Conclusions:**

A comprehensive data collection system in primary care is possible to achieve with partnerships that balance researcher, clinical, and policy maker contexts. Engaging practices as valued community members and independent business owners requires significant time, and financial and human resources. An integrated knowledge translation and exchange approach provides a foundation for continued dialogue, exchange of ideas, use of the information produced, and recognises recruitment as part of an ongoing cycle.

## Background

High performing primary care is foundational to achieving the triple aim of health reform—better health, improved patient experience, and more affordable costs [1]. Bodenheimer and colleagues [2] suggest 10 building blocks of high-performing primary care; Data-driven improvement was one of four foundational building blocks necessary before achieving success in the higher order blocks. Yet, much of what we know about high performing primary care is based on analyses using health administrative data [3–6] and chart audits, [7, 8] not data from patients, clinicians, or the practices. Moreover, across Canada and internationally we have poor infrastructure to regularly collect survey data from primary care practices to supplement the administrative data. Primary health care clinicians in Canada have historically low (2–21%) and declining response rates for survey research [9–11], comparable to other countries [12]. Common barriers include time constraints, disruption to clinic flow, competing research, and a fear of performance evaluation and its consequences [13–15]. Additionally, the environment for collecting data from multiple sources (e.g. administrative, surveys) across different organizations and regions remains challenging given the diverse custodians of data, multiple ethics review committees and privacy impact assessments.

In order for practices and jurisdictions to achieve data driven improvement, data systems that track clinical (e.g. diabetes management), operational (e.g. continuity of care and access) and patient reported experiences and outcomes are needed [2]. If there is to be more routine and widespread practice-based data collection an integrated knowledge translation and exchange (KTE) strategy is needed in order to develop greater research capacity and goodwill in future projects by meaningfully engaging participants in primary care research [16]. Improvement towards high performing community-based primary health care (CBPHC) requires those who can influence change or take action on indicators have accurate and meaningful measurement information for reporting [17–31]. Involving patients [32], clinicians [33], and practices [33] in contributing to meaningful measurement in CBPHC is essential to driving improvement. As a guide for others interested in developing data systems that can drive improvement and to support the further development of these approaches, we present strategies for recruitment of practices using the CollaboraKTion Framework [34, 35]. The CollaboraKTion Framework is a KTE approach sensitive to context and is focused on establishing partnerships with organizations and individuals.

The purpose of this paper is to report on lessons learned in working with CBPHC practices to recruit and collect data. We add new information to the literature by: 1) examining the variable costs for carrying out primary care practice-based surveys; and 2) sharing lessons learned regarding the level of engagement required for recruitment of practices in primary care.

## Methods

**Fig. 1:**
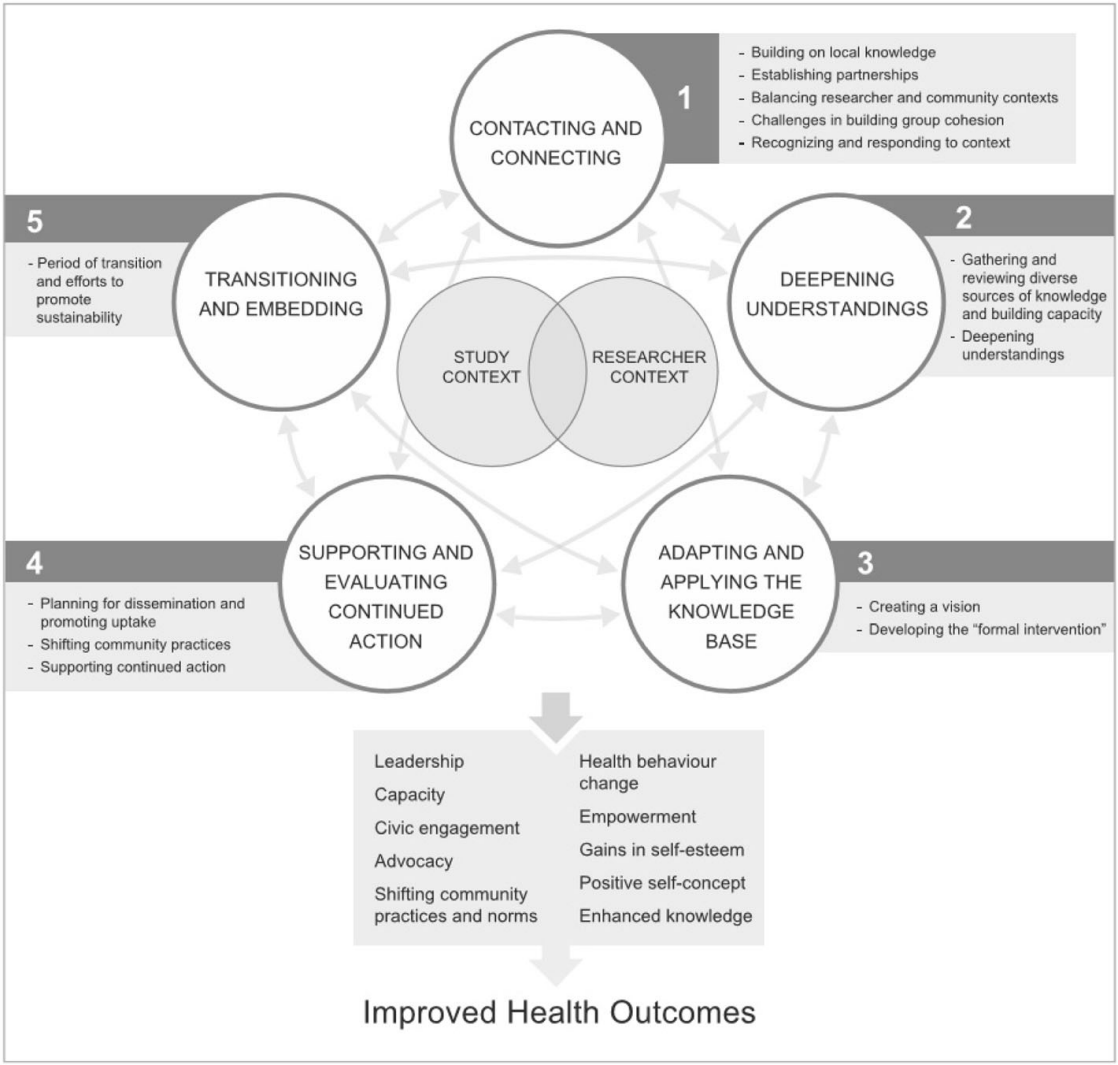
CollaboraKTion Framework (reproduced with permission from authors) [33]

Given the historical difficulty of practice-based recruitment, we used an integrated (KTE) approach to engage with potential participants. While we did not explicitly use any one integrated KTE approach during practice recruitment and data collection, the CollaboraKTion framework provides a well-aligned approach to reporting our diverse recruitment strategies. The CollaboraKTion framework is an expansion of Kitson’s CoKT framework, which accounts for researcher and community contexts that converge on a set of 5 iterative activities: 1) Contacting and Connecting; 2) Deepening Understandings; 3) Adapting and Applying the Knowledge Base; 4) Supporting and Evaluating Continued Action; and 5) Transitioning and Embedding (Fig. 1) [34, 35].

TRANSFORMATION is a cross-sectional study on improving the science and reporting of CBPHC performance in three Canadian geographic regions: Fraser East, British Columbia (BC); Eastern Ontario Health Unit, Ontario (ON), and Central Zone, Nova Scotia (NS). As part of the larger study, we carried out a practice-based survey. Since the study remains ongoing, we report here on practice recruitment activities. Physician, staff and patient participation in data collection for ongoing CBPHC performance measurement and reporting is critical, making the CollaboraKTion framework a useful roadmap for implementing a comprehensive data system that could be used to drive CBPHC performance.

### Participants

In Fraser East, BC, there were 164 providers (family physicians and nurse practitioners) working in 58 practices; in Eastern Ontario Health Unit, ON, there were 190 providers in 63 practices; and in Central Zone, NS, there were 190 providers in 123 practices. Once the list of practices and providers was verified by each region’s stakeholder advisor group, we recruited between 22 and 39 eligible primary care practices via individual primary care providers within the region. Our sample size calculation required a minimum of 15 practices per region to detect minimal differences of 2–5% across jurisdictions in two main patient-level performance indicators, access and continuity of care, using a Bonferroni-corrected two sided alpha level of 0.017 to account for multiple pair-wise comparisons. For these calculations, we used estimated standard deviations and intraclass correlation coefficients from data collected in the Comparison of Primary Care Models in Ontario study [4]. These calculations take into account that patients who share the same provider may be more similar to each other and may have similar primary care experiences, and that different providers from the same practice may not practice as independently from each other as providers in different practices. A maximum of five physicians/ nurse practitioners per *independent* practice were eligible to participate. Based on previous work [36], practices were considered independent if they did not share more than four of the following five criteria: 1) office space; 2) staff; 3) expenses; 4) patient records; and 5) on-call duties. Physicians and nurse practitioners were eligible to participate if they met the following criteria: 1) must be part of their current practice for at least 1 yr; 2) identify that practice as their “principal clinical practice”; and 3) practice comprehensive, all-ages primary care and not limit their practice to a special focus such as sports medicine, emergency medicine, palliative care, or psychotherapy (i.e., the special focus does not comprise more than 20% of their total practice time).

### Recruitment

Providers were initially informed about the study through a letter or email sent by local decision-makers. Research coordinators in each region then mailed out a recruitment letter to physicians with an expression of interest mail-back card enclosed. Those who sent back expression of interest cards were contacted immediately by the research coordinators, whereas those practices who did not send back an expression of interest were contacted by phone after ten business days. We expected to send up to three follow-up reminder emails/phone calls but left our recruitment protocol open to more contacts if needed. For consenting practices, organizational leads were asked to complete one organizational survey, and one to five physicians or nurse practitioners completed a provider survey in each practice. To minimize burden on providers, this survey was kept as short as possible (e.g. number of years practicing, age range, gender). All staff were also asked to complete one Team Climate Survey [37, 38]. Once practices agreed to participate, we then recruited a consecutive sample of their patients (a minimum of 20 per practice) to fill out a paper-based patient experience survey. Patients were eligible to participate if the following criteria were met: 1) aged 18 years and over; 2) have been with their current provider for at least 1 yr; and 3) able to complete the survey in either English or French.

Data were collected between 2014 and 2016. Provider and organizational surveys were collected using REDCap (Research Electronic Data Capture) [39] or paper surveys. A survey administrator distributed patient surveys in primary care practice waiting rooms. Written informed consent was obtained from all clinicians and patients. Patients were specifically asked for written consent to: a) participate in the survey, b) have their survey data linked to the administrative data, c) be contacted again for further related studies. All procedures were approved by the Behavioural Research Ethics Boards at Fraser Health, University of British Columbia, Ottawa Health Science Network, Bruyère Continuing Care, and the Nova Scotia Health Authority.

### Data sources & analysis

Data for this report of study recruitment methods were derived from qualitative sources including: study team meeting minutes; memos/notes from survey administrators regarding their interactions with practice staff, providers, and patient participants; and stakeholder meeting minutes. Data were also derived from spreadsheets with numbers for participant eligibility, responses, and survey completions and from time and cost tracking for recruitment and survey administration.

All notes were electronically documented. We used the CollaboraKTion framework steps to guide our analysis. Notes were read several times and coded by two members of the research team according to the first three steps: contacting and connecting; deepening understandings; and adapting and applying the knowledge base. Codes were then organized into themes; initial coding and author agreement was reached through an iterative process of discussion and returning to the data.

We calculated participation and completion rates for the patient surveys using the Wong et al. [12] approach to calculating the different rates for comparability with existing literature on CBPHC performance measurement. This method tracked the participation process, allowing the elucidation of “steps” and their respective attrition rates. The project had one research coordinator per region, which represents our fixed costs of carrying out this work. We also calculated a detailed breakdown of variable costs required for practice recruitment and survey implementation in each province.

## Results

A total of 87 practices participated in the study (*n* = 22 in BC; *n* = 26 in ON; *n* = 39 in NS). Future analyses will be conducted using administrative health data to better understand how our sample compares to the larger population studied and how our sample compares to another practice-based survey sample conducted for the Quality and Costs of Primary Care Study [12].

### Recruitment procedures

In recruiting practices, the first three CollaboraKTion activities, Contacting and Connecting, Deepening Understandings, and Adapting and Applying the Knowledge Base, and their associated processes were most pertinent to our participant and community engagement strategy. As CBPHC performance measurement and reporting continues beyond the end of the study and scales up in the study regions, the other two CollaboraKTion activities, Supporting and Evaluating Continued Action, and, Transitioning and Embedding, will become more relevant with continued re-iteration through the other three activities. Table 1 provides an overview of the specific actions we took in engagement during our recruitment and data collection phase. In keeping with the iterative nature of the CollaboraKTion framework, the described actions did not necessarily happen chronologically as presented. Extracting the qualitative data and our spreadsheets on recruitment training was key for our discussion of the costs associated with recruitment (Excel tracking sheets) and the different strategies for engagement in each region (meeting minutes and data collection notes).

**Table 1:** Variation in approaches across sites

	General approaches	Regional additions/variation		
		BC	ON	NS
Practice Recruitment	Regional study advisory stakeholder committee	Yes – comprised of lead physicians and executive directors from the Chilliwack, Abbotsford and Hope Divisions of Family Practice^a^, other health professionals, patients, and policy makers (*n* = 12)	No - Email correspondence with local physicians and policy makers for advice (*n* = 6)	Yes – comprised of local physicians, other health professionals, patients, and policy makers (*n* = 12)
	Engagement with local organizations	Partnership with Divisions of Family Practice. Meetings with Doctors of BC and General Practice Service Committee	Presentations to the Association of Family Health Teams, Health Quality Ontario, and the Ministry of Health and Long-Term Care	Meetings with the Nova Scotia Health Authority Department of Family Practice, Department of Health and Wellness, Provincial Primary Health Care Teams Operations Networking Group
	Presence at physician-attended events	Standalone TRANSFORMATION events hosted by each Division of Family Practice in the study region Billing Workshop for physicians	Local Health Integration Network (LHIN) conference^a^	Health authority’s Career Development Event Health authority’s Department of Family Practice Forum
	Peer-to-peer practice recruitment	Three peer-to-peer recruiters.	Four geographically-dispersed peer-to-peer recruiters	One peer-to-peer recruiter
	Demonstrate study relevance	Offered practice-based portrait of study findings 1-page brief created, with preliminary data as it is available, for peer-to-peer recruiters to use Standalone TRANFORMATION catered dinner to share preliminary data	Practices already receive practice-based feedback from provincial organization	Offered practice-based portrait of study findings
Patient Recruitment	Localized survey implementation	Hired localized survey administrators (SAs) Hired Punjabi-speaking SA for practices with high proportion (> 50%) of Punjabi-speaking patients	Did not hire localized SAs because researchers did not have sufficient ties to research assistants in the study region Surveys available in both English and French	Hired localized SAs Surveys only available in English
	Token of appreciation	$10 coffee gift card to patients	No gift card offered	$5 coffee gift cards to patients

^a^Divisions of Family Practice are groups of family physicians that work to achieve common health care goals within communities [49]; Local Integrated Health Networks (LHINs) are community-based health authorities that plan and coordinate local health care services [50]

### Contacting and connecting

#### Building on local knowledge

Determining the landscape of CBPHC practices in each region was challenging due to a lack of centralized sources that describe regional primary care practice structures. We conducted an extensive investigation in each region on the number and types of practices present. For example, in Ontario, we worked with the regional health authority called the Champlain Local Health Integration Network (LHIN) to identify practicing family physicians and nurse practitioners. In all regions, we used other publicly available sources such as information from the College of Family Physicians of Canada, provincial Colleges, local health authorities, and Google.

We hired survey administrators, who had local knowledge of the communities within each region to administer the patient surveys at each participating clinic. Their presence allowed the research team to work directly with the practice staff and patients. In NS and BC, Regional Stakeholder Advisory Committees were formed to advise on all aspects of the study, including tailoring recruitment approaches in each region. These committees included local experts who held a variety of roles, including patients, physicians and decision-makers, in a variety of clinical, academic, policy, and other decision-making contexts. Ontario took a different approach by individually engaging physician opinion leaders representative of their study region. Together, team members and clinicians strategized how to avoid overwhelming practices by being aware of potentially competing “asks” on their time. For example, in NS, we were mindful not to recruit during times when several other local asks for practice participation in research were in progress. When the study team noted enrollment of new practices coming to a standstill, all regions employed local physician peer-to-peer practice recruiters. These paid physician recruiters were identified through existing relationships with the research team and were chosen largely based on their connections to the local physician community.

#### Establishing partnerships

Having previous experience in physician and patient recruitment but minimal or modest existing networks of relationships within the study regions, the team devoted substantial resources to engage practices in recruitment. Over a series of regional advisory meetings, 13 in BC and 7 in NS, we listened and learned about how primary care was delivered in each jurisdiction and the political tensions in conducting research or trying to measure performance of primary care. In addition, we established and held meetings of an International Stakeholder Advisory Group consisting of international experts in primary care research, utilizing one of the meetings to learn from the expertise of the committee to better understand recruitment issues. To foster ongoing relationships, we invited several local and provincial decision-makers from each study region to annual face-to-face full team meetings and to other team meetings within the region. In BC, we partnered closely with three Divisions of Family Practice, who provided guidance for tailored recruitment in each Division. In exchange, we provided the option to give data back to the Divisions (with participants’ consent). We also made presentations to other relevant physician-attended community organizations and initiatives, locally and beyond (See Table 1).

#### Balancing researcher and community (clinical) contexts

The divide between academic and clinical contexts emerged early in our recruitment over the use of the term “independent” practices. For clinicians, the term independent was tied to notions of being independently responsible for their medical practice, whereas for the purposes of our study, an independent physician shares up to four of the following features: space, staff, on-call duties, records, and finances [36]. In BC, decision makers and clinicians had particular challenges with this definition as it didn’t fit with their conceptualization or realities of practice (physicians who had independent panels of patients but were working in large groups). However, we had designed and funded recruitment based on assumptions of how family physicians were practicing (mostly single providers) [36]. Given our mutual authentic engagement on this grant, clinicians, health authority partners and researchers were able to appreciate each other’s contexts and carry on with recruitment and data collection. Scientific rigor and recruitment according to our eligibility was maintained. It was also agreed that the research team provide participating practices with a report on their individual performance, if requested.

The research team was able to recruit some practices on their own. However, there were competing demands for time by clinicians on our team. For example, one clinician on our team also was a senior administrator in ON. He was enthusiastic about the goal of our study (CBPHC performance measurement and reporting) and originally agreed to help recruit practices and promote the study and its objectives but was unable to commit the time to fulfill his role due to ongoing and emerging priorities in his regular position.

In order to balance research and clinical contexts we used two additional recruitment strategies. First, we employed physician recruiters, 3 in BC, 4 in ON, and 1 in NS, who were more likely to understand the day-to-day practice reality and connect directly with potential physician participants. Physician peer recruiters were successful in recruiting “harder to reach” CBPHC practices. These were practices in which the research coordinator had made three follow-up phone calls but still had no answer from the practice. Research coordinators spent approximately 30% or more of their time on recruitment for three to 4 mos before the peer-to-peer recruiters were employed to assist with practice recruitment. All recruiters, except for one, were selected largely based on their pre-existing relationships with many of the practices in each region. Second, in order to keep data collection minimally disruptive for practices, we hired survey administrators who could work with each practice’s population. Survey administrators worked in English (all regions), Punjabi (BC), and French (ON).

To address the known barriers regarding provider compensation for participation in research, we offered a number of incentives for participation. Upon completion of the provider and organizational surveys, practices received an honorarium in recognition for their time ($250 in NS and BC; $500 in ON). Offering additional funds to providers in ON was a local strategy developed after the initial protocol. Family physicians were also offered Continuing Medical Education credits. In BC and NS, the practice staff who worked with our survey administrator, as well as patients who completed the patient survey, were provided with coffee cards (e.g. Tim Horton’s, Starbucks) as a token of appreciation.

In order to balance the clinical contexts, researchers built relationships with each consenting practice in order to find dates and times for recruiting potential patients. On practices’ requests, a poster for the lunch or staff room was provided to each practice to inform and/or remind staff when patient recruitment will take place. On the date set by practices, a survey administrator from TRANSFORMATION recruited up to 20 consecutive patients for each participating physician/nurse practitioner. A key relationship was between the survey administrator and the office staff. Together, they went over procedures for recruitment and data collection. Practice staff were provided with a script to introduce patients to our on-site survey administrator. The survey administrator then explained the study, assessed eligibility, and obtained informed consent from participants. Patients were also asked if they would like to participate in related studies (e.g. focus groups and/or deliberative dialogues), consent to link their survey data to health administrative data via patient health card numbers, and consent to a follow-up phone or email survey of approximately five questions.

#### Challenges in building group cohesion

Our recruitment and engagement strategy was diverse. It did not require all stakeholders to totally agree on our processes across or within the regions. We did, however, encounter challenges with group cohesion when it was desired. Neither ON nor NS had purposeful organizations of CBPHC practices such as divisions of family practice in BC (See Table 1). In ON, the large geography of the study region paired with the concentration of the investigators and study staff in a single metropolitan area outside of the study region prohibited the formation of a cohesive regional stakeholder advisory committee. Instead, ON had 6 study advisors whom we consulted on an ad hoc basis. In the other regions, we struggled to sustain committee participation and had particular trouble finding meaningful ways to engage patient members. For example, in NS, we sent out an open call for patient representatives through a local volunteer recruitment website. We recruited three patient representatives to the committee. While the study team did not expect the patient representatives to have very much knowledge of the study topic prior to committee participation, we had hoped that orientation to the topic over multiple meetings would spur interest, but inconsistent attendance at meetings and the prioritization of personal agendas only tangentially related to the study topic were barriers to meaningful and productive involvement. More targeted patient recruitment approaches in BC, through an existing patient engagement organization, yielded representatives better equipped to engage with our study topic, as this organization matches patient representatives to opportunities suited to their interests.

### Deepening understandings

#### Gathering and reviewing diverse sources of knowledge and building capacity

We aimed to build our own skills in the contexts of our study regions and share our learnings with the broader community to raise awareness and capacity for CBPHC performance measurement and reporting. We began the study with an understanding of the challenges of provider and patient recruitment from several previous studies and initially drafted our recruitment strategies using best practices found in the literature. We looked to our regional committees and advisors to help amend and contextualize our procedures and to communicate back out with their communities. Similarly, we met with a number of provincial organizations, which allowed for a two-way dialogue. We also met with several other provincial, national, and international stakeholders, including our international scientific advisory committee, to gather advice and share our own findings to the international community. In several of these engagements, we shared our surveys to inform tool development for other performance measurement and related initiatives.

#### Deepening understandings

Meeting recruitment targets hinged on making our work relevant to potential participants and deepening their knowledge of CBPHC performance measurement and reporting. We presented to a large number of national, provincial, and regional stakeholders to spread the word about performance measurement and the value of our study. Our regional advisory committees identified the need for practice feedback and advised us on how to proceed. In the spirit of reciprocity, we offered to produce Practice Portraits for participating practices that would contain practice-level information from the suite of surveys, linked to health administrative data.

### Adapting and applying the knowledge base

#### Creating a vision

Unlike the prescribed use of Co-KT and CollaboraKTion frameworks, our vision to create conditions conducive to comprehensive CBPHC performance measurement with sufficient response rates in the study regions was determined a priori*.* However, through extensive engagement with relevant communities in each region, we integrated their knowledge to iteratively refine the vision. We identified and posited solutions to barriers we faced specifically in the recruitment and engagement strategy and generally in promoting CBPHC performance measurement and reporting.

#### Developing the information system

In our study, we introduced CBPHC performance measurement and reporting approaches, and through our iterative recruitment strategies, tested their sustainability and acceptance in the study regions. Our methods manifested differently in each study region in reaction to contacting and connecting with local stakeholders and reciprocally deepening understandings of the local researcher and clinical realities. To overcome challenges in recruiting practices and patients, we implemented a “learning system” approach, using a variety of strategies with localized, contextual variations. A core set of the research team (co-principal investigators and staff) met weekly to deal with any issues arising during the course of recruitment and data collection; we continuously revised our methods, within the protocol limits, by learning from our sample.

### Practice recruitment

**Fig. 2:**
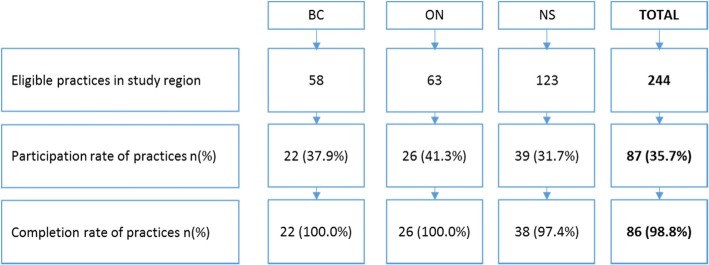
Participation rates of practices. Participation rates were calculated as the number of practices that were recruited to the study divided by the number of eligible practices in the region, all of which were invited to participate. Completion rates were calculated as the number of practices that returned completed (at least 85% of questions answered) organizational surveys divided by the total number of participating practices. British Columbia (BC), Ontario (ON), Nova Scotia (NS)

Practice participation and completion rates for all three study regions are presented in Fig. 2. Participation rates was similar (average was 36%) across the study sites and completion rates were high (99%) once practices agreed to participate.

### Patient recruitment

**Fig. 3:**
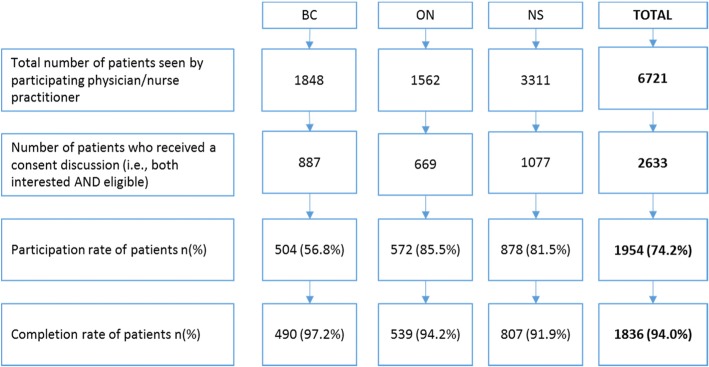
Participation and completion rates for patient recruitment. Participation rates were calculated as the number of patients who consented to participate divided by the number of interested and eligible patients. Completion rates were calculated as the number of patients who completed 85% or more of the survey questions divided by the total number of patients who consented to participate. British Columbia (BC), Ontario (ON), Nova Scotia (NS)

Patient participation and completion rates for all three study regions are presented in Fig. 3. Participation rates in BC were substantially lower than in the ON or NS sites. In part, this may have been due to the involvement of front office staff. In BC, the receptionist only introduced patients to the survey administrator (SA) if the patients were both interested and eligible, whereas the front office staff in the other provinces were encouraged to send all interested patients to the SA so that the SA could directly screen the patients for eligibility. While all provinces had intended on the receptionist screening for patient eligibility, we found that the staff in ON and NS were too busy to do this additional step. It is possible that the BC front office staff may have informally screened patients for eligibility in a way that some patients who could have been eligible and interested in participating were not told about the study. We learned that the front office staff appreciated being as minimally disrupted as possible, and in cases such as ours where there was an opportunity for research staff to be on site during patient survey recruitment and data collection, it may be advantageous to screen for patient eligibility directly.

### Time and variable costs of practice recruitment and data collection

The variable costs required to recruit practices and administer the patient surveys ranged from $1503 to $1792 and are summarized in Table 2. In BC and NS, the survey administrator was embedded within the community so less time (and expenses) was spent on travel. In ON, the survey administrator was based at the university and spent more time in travel. The lower practice recruitment costs in the areas of peer recruiter and community engagement in NS are likely due to the fact that they had the highest number of eligible practices in their study region and they were able to recruit more practices. Researchers in NS also had strong ties to decision maker leads and existing research relationships with many of the physicians in the region and the peer recruiter for NS stood out in that they were particularly capable of quickly recruiting practices, largely due to pre-existing relationships with physicians in the community.

**Table 2:** Time and variable costs for practice-based survey data collection

	BC	ON	NS	All sites
Number of practices	22	26	39	87
Mean number of data collection days per practice (SD)	2.32 (1.86)	3.50 (1.50)	4.95 (2.89)	3.89 (2.43)
Honoraria for practices/providers	$5500	$13,000	$9750	$28,250
Tokens of appreciation for staff and patients	$5240	$0	$5645	$10,885
Physician peer recruiters	$5916	$3360	$2145	$11,421
Community engagement for implementation	$7886	$3805	$3641	$15,332
Survey administrators for patient survey implementation	$14,894	$25,938	$37,441	$78,273
Total	$39,436	$46,103	$58,622	$144,161
Average costs per practice	$1793	$1773	$1503	$1657

The cost of Survey Administrators includes travel time and mileage ($25 CAD/hour, $0.40 CAD/km), time spent in practice, meals, and accommodation. Cost of community engagement includes honoraria for committee participants, meeting costs, travel for external presentations, and bringing decision makers to full team face-to-face meetings

Each region strategized practice recruitment expenditures differently. Although recruiting practices was the main concern for success of our study and was also the largest recruitment expense, we did funnel some funds into patient recruitment. BC and NS offered coffee cards to front office staff and participating patients. Given the lack of existing relationships between researchers and potential practice participants and the known time conflicts of providers in the ON study region, the research team in ON decided to offer practices a higher honorarium amount than the other provinces ($500 vs. $250). This meant that they did not have the funds to offer gift cards to patients. BC also used funds for community engagement and peer recruiters. Across all sites, recruitment funds were targeted in ways most appropriate to their given contexts.

## Discussion

Recruiting primary care practices and gathering data from clinicians, staff, and patients, who are considered to be in the best position to report on specific core attributes of primary care, is feasible. We outlined our challenges and successes in practice recruitment, as well as the variable costs associated with recruitment and data collection, taking local contexts into account and engaging the community through a KTE approach. Across the study we also had fixed costs of a research coordinator per region. The generalizability of our results is enhanced in that we took our findings from three different Canadian health regions across BC, ON, and NS. As part of our KTE approach, we emphasize the necessity to arrive at a tailored approach to recruitment, through a process of community engagement and adapting to the needs of each region. Much time was spent within the three jurisdictions on 1) contacting and connecting with primary care practices; 2) deepening understandings of each other as service delivery clinicians and researchers; and 3) adapting and applying our common and distinct knowledge bases to create a common vision and develop a primary care information system.

Insights from our study show that multijurisdictional research initiatives may require different strategies in each region to achieve similar recruitment rates. Flexibility of protocols and recruitment budgets are key, and researchers should seek local inputs throughout research processes. Researchers should note that tailoring approaches may result in project delays when navigating the complexities of multijurisdictional research (e.g. multiple research ethics boards, different requirements for data privacy, etc). Through strategies such as partnering with local advisors and decision makers and hiring peer-to-peer recruiters, the ‘contacting and connecting’ theme was the most immediately integral of the three themes to recruiting practices. However, each of the three integrated KTE themes were integral to our current and future success in the long term by furthering buy-in to our study’s objective in a way that was meaningful and useful to participants. Using this multi-pronged approach to recruitment, we were able to recruit 32–41% of all eligible practices in a region. This is an achievement, particularly considering we were asking a lot out of practices (four different types of surveys, patient data collection over multiple days, etc.). Reasons given for not participating were similar to what has been reported in past work: time constraints, disruption to clinic flow, fear of performance evaluation and its consequences [13–15] and that survey administration with their specific patient panels (e.g. high number who were Indigenous) was not appropriate.

A comprehensive data collection system in primary care that can drive improvement is possible to achieve with partnerships that balance researcher, clinical and policy maker contexts. There are challenges building group cohesion and recruiting independently owned and operated primary care practices. Working with practices ought to take a two-way learning approach. Engaging with practices and building relationships between researchers and clinicians take time. Importantly, implementation of findings requires capturing their minds, hearts and attention with the significance of the work being proposed. By re-orienting our approach from project-based – entering practices only to collect data once and then leaving – to a KTE approach for recruitment, we can engage with practices and other stakeholders iteratively over time. Our approach is likely too expensive for widespread use throughout primary care. However, we suggest this framework could be used more broadly in primary care in thinking about how to create ongoing relationships for purposes of healthcare learning at the health authority level. In particular, it is helpful for considering front-end strategy development, relationship building, and flexible recruitment implementation. Primary care practices are an essential part of each community. Engaging them as valued community members and independent business owners requires significant time and resources.

Indeed, several strategies have proven successful in improving physician response rates to survey research, which embed elements of a KTE strategy. In addition to modified versions of the proven Dillman approach [40], many recruitment protocols focus on engagement strategies targeted to local contexts. Regional factors vary, requiring recruiters to adapt tailored and iterative recruitment approaches [41]. Clinician-to-clinician recruitment has been used successfully to improve physician response rates [13, 41, 42], where familiarity between the recruiter and participant improves rapport [43]. In particular, using recruiters who are known to prospective participants allows them to be cognizant of and responsive to local conditions that may affect participation [44]. Similarly, endorsement from professional organizations synchronized with that from local champions have also been shown to assist in recruitment [41, 42, 45]. These strategies align with our peer-to-peer approach, which had variable success across the study regions.

A lesson learned from our work is the value of budgeting for flexibility in recruitment approaches and that some regions may need more time and resources than others. We show how we flexibly use recruitment funds, channeling them differently in the three regional contexts to achieve similar response rates. Ontario had the highest recruitment rate for both practices and patients. This region offered more funds to the physician than the other regions and did not offer patients or practice staff a gift card. Given that the practices, not the patients, were the most challenging to recruit, ON’s strategy for higher physician compensation may have contributed to the better responses. Another lesson learned is to be mindful and understand the interplay of relationships within each local context. For example, some of the providers in our regions expressed disinterest in our study due to tensions with our local partners (provincial ministries and regional health authorities). It is helpful to keep these types of tensions in mind when working with local partners.

The literature on recruitment strategies is focused on pre-data collection recruitment and does not capture post-study engagement. Steps four and five of the CollaboraKTion framework [34], supporting and evaluating continued action, and, transitioning and embedding, describe continued engagement throughout intervention development, implementation, and beyond. In the context of our study’s research topic, CBPHC performance measurement and reporting, recruitment was conceptualized as an ongoing and iterative KTE activity. Building a learning research environment, where practices’ participation comes with access to expertise and opportunities to learn about and shape performance reports useful to them and recruitment for subsequent studies facilitated by the involvement of past participants, could be a solution to historically low primary care participation.

The recruitment and data collection strategy of TRANSFORMATION adds to the existing literature on practice-based engagement, above and beyond physician recruitment. The regional stakeholder advisory committees in BC and NS are an important component of the integrated KTE approach. These committees will assist in carrying out the CollaboraKTion framework steps of supporting and evaluating continued action and transitioning and embedding knowledge. Our model of connecting with community partners and stakeholders will also continue as the study progresses through presentations, meetings, and stakeholder feedback on the products of our study.

This work is limited in that we worked with three geographic areas within three provinces. While these learnings may not apply to all jurisdictions, those with similar contexts may find this information useful. Our “intervention” of performance measurement and reporting was researcher driven and not as participatory in some respects, such as how a practice was defined. Ideally, the community and researchers would jointly agree on all aspects of the research project, including the intervention, before undertaking the study. Also, given the tendency of practice-based surveys to have certain response biases [46, 47], an important part of our future analysis will be to assess for the representativeness of our survey sample.

A community-based engagement approach can be expensive. Developing trust and lasting relationships requires time, energy, and financial resources. Managing the regional budgets required localized approaches and trade-offs. For example, in ON, higher per-practice honoraria were offered instead of tokens of appreciation for patients. In addition to the added costs of the intensive hands-on recruitment and survey administration strategies, such as community engagement, hiring peer recruiters, and hiring local highly qualified survey administrators, practices still expect to be compensated for their participation in research activities [13, 14, 48]. One place to decrease costs and potentially increase the recruitment rate of practices may be to administer patient surveys using electronic approaches and automation of patient recruitment. Automating patient, provider, and organizational survey administration could assist if CBPHC practices routinely need to collect data or if there was demand for a more widespread (e.g. multiple health authorities, provinces, etc.) practice-based survey to inform delivery of primary care.

## Conclusions

The CollaboraKTion framework was a good match for our study in that we were able to use the framework to more comprehensibly organize our discussion of our recruitment activities. There are some key lessons which could assist in future primary care work. First, collecting data from patients, clinicians, and teams requires community-based engagement strategies as early as possible. The roles of the office staff (receptionist, office manager) are important to successful recruitment and data collection. While some automated extraction of data from primary care is possible (e.g. via electronic medical records or telephone robots), the need to engage practices and patients will continue to be necessary. Second, our practice recruitment rate (36%) remained similarly low, and therefore likely lacks external generalizability to the regions, across the three sites despite context-specific strategies and working with different primary care organizations such as the Divisions of Family Practice in BC. Without a clear need to participate in practice-based surveys (e.g. requirement to report on patient experiences in order to receive funding), working with CBPHC practices remains challenging. More work is needed to increase participation rates of CBPHC practices within regions. Third, engaged CBPHC practices, however, play an important role in developing, contributing to and implementing research, quality initiatives and system change. Co-created recruitment and data collection strategies in addition to a feedback loop between researchers, clinicians, policy makers and patients can move us in the direction of a learning health system in primary care. Finally, an integrated KTE approach provides a foundation for continued dialogue, exchange of ideas, use of the information produced, and recognises recruitment as part of an ongoing cycle that supports the need to develop our culture of learning in CBPHC.

## Abbreviations

BC: British Columbia
CBPHC: Community-based primary health care
KTE: Knowledge translation and exchange
LHIN: Local Health Integration Network
NS: Nova Scotia
ON: Ontario
SA: Survey administrator
